# CyTOF-Enabled Analysis Identifies Class-Switched B Cells as the Main Lymphocyte Subset Associated With Disease Relapse in Children With Idiopathic Nephrotic Syndrome

**DOI:** 10.3389/fimmu.2021.726428

**Published:** 2021-09-21

**Authors:** Miguel Fribourg, Michela Cioni, GianMarco Ghiggeri, Chiara Cantarelli, Jeremy S. Leventhal, Kelly Budge, Sofia Bin, Leonardo V. Riella, Manuela Colucci, Marina Vivarelli, Andrea Angeletti, Laura Perin, Paolo Cravedi

**Affiliations:** ^1^Department of Medicine, Icahn School of Medicine at Mount Sinai, New York, NY, United States; ^2^Nephrology, Dialysis and Transplantation Unit, Istituto di Ricovero e Cura a Carattere Scientifico (IRCCS) Istituto Giannina Gaslini, Genova, Italy; ^3^Dipartimento di Medicina e Chirurgia Università di Parma, Unitá Operativa (UO) Nefrologia, Azienda Ospedaliera-Universitaria Parma, Parma, Italy; ^4^Division of Nephrology, White Plains Hospital, White Plains, NY, United States; ^5^Center for Transplantation Sciences, Division of Nephrology, Massachusetts General Hospital, Harvard Medical School, Boston, MA, United States; ^6^Renal Diseases Research Unit, Genetics and Rare Diseases Research Area, Bambino Gesù Children’s Hospital IRCCS, Rome, Italy; ^7^Division of Nephrology, Department of Pediatric Subspecialties, Bambino Gesù Children’s Hospital IRCCS, Rome, Italy; ^8^Gabriel Organization for All Renal Research (GOFARR) Laboratory, Children’s Hospital Los Angeles, Division of Urology, Saban Research Institute, University of Southern California, Los Angeles, CA, United States

**Keywords:** nephrotic syndrome, B cell, T cell, predictor, relapse, immune phenotype

## Abstract

B cell depleting therapies permit immunosuppressive drug withdrawal and maintain remission in patients with frequently relapsing nephrotic syndrome (FRNS) or steroid–dependent nephrotic syndrome (SDNS), but lack of biomarkers for treatment failure. Post-depletion immune cell reconstitution may identify relapsing patients, but previous characterizations suffered from methodological limitations of flow cytometry. Time-of-flight mass cytometry (CyTOF) is a comprehensive analytic modality that simultaneously quantifies over 40 cellular markers. Herein, we report CyTOF-enabled immune cell comparisons over a 12-month period from 30 children with SDNS receiving B cell depleting therapy who either relapsed (n = 17) or remained stable (n = 13). Anti-CD20 treatment depleted all B cells subsets and CD20 depleting agent choice (rituximab *vs* ofatumumab) did not affect B cell subset recovery. Despite equal total numbers of B cells, 5 subsets of B cells were significantly higher in relapsing individuals; all identified subsets of B cells were class-switched. T cell subsets (including T follicular helper cells and regulatory T cells) and other major immune compartments were largely unaffected by B cell depletion, and similar between relapsing and stable children. In conclusion, CyTOF analysis of immune cells from anti-CD20 antibody treated patients identifies class-switched B cells as the main subset whose expansion associates with disease relapse. Our findings set the basis for future studies exploring how identified subsets can be used to monitor treatment response and improve our understanding of the pathogenesis of the disease.

## Introduction

Pathogenesis of idiopathic nephrotic syndrome (INS), the most frequent pediatric glomerular disease (1), remains poorly understood. Familial forms of INS are characterized by genetic abnormalities (2, 3), whereas non-genetic INS forms are hypothetically immune-mediated, possibly *via* unknown circulating permeability factors (4). The prevailing hypothesis, enshrined by a classic 1974 Shalhoub article in *The Lancet*, posits INS as a T cell disorder (5). Accordingly, first-line treatment for INS are corticosteroids with a complete remission rate of about 80%. However, after initial responses, 40%–50% of patients can experience frequent relapsing-remitting episodes and become steroid-dependent or, eventually, steroid-resistant (6–8).

Pathogenic contribution from B cells was suggested in 2006, when Pescovitz et al. described the case of a 7-year-old boy with post-transplant focal segmental glomerulosclerosis recurrence [FSGS, thought to represent an evolution of minimal change disease, MCD (9)] who underwent remission after he received B-cell depletion to treat post–transplant lymphoproliferative disease (PTLD) (10). Subsequently, numerous case reports and clinical trials documented efficacy of B cell depleting therapies in inducing/maintaining long-term remission (11–14). Some reports document temporal correlation between B cell reconstitution and relapse (15, 16), while other patients remain in remission despite B cell recovery (17, 18). This formed the basis for our studies testing the hypothesis that differential immune cell frequencies during B cell recovery underlie differences in relapsing *versus* non-relapsing patients after B cell depletion.

**Figure 1:**
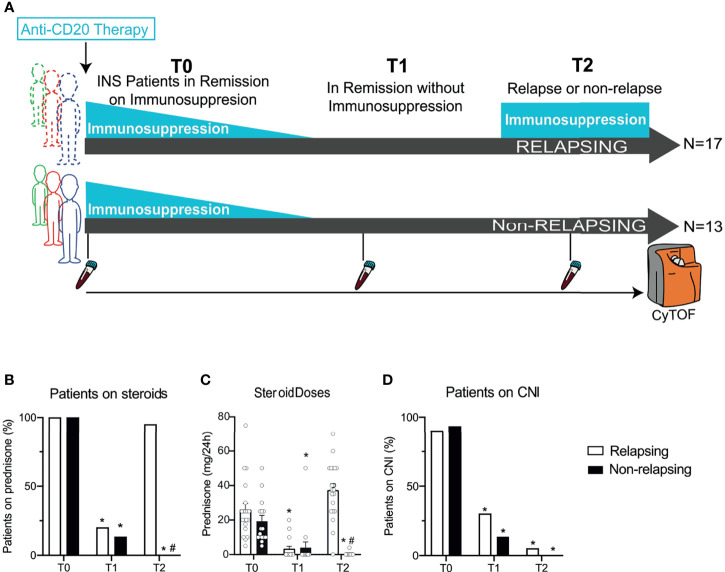
Serial high-dimensional profiling of INS patients and concomitant immunosuppressive treatments. **(A)** Study design of thirty children with steroid-dependent nephrotic syndrome in remission with steroids ± calcineurin inhibitors who received either rituximab (RTX) or ofatumumab (OFA) and then underwent withdrawal of immunosuppression in 3 months. After withdrawal, 17 patients developed a relapse, while 13 stayed in remission. Blood was collected before RTX or OFA therapy (T0), after immunosuppressive withdrawal (while still in remission; T1), and at the time of relapse (or at the same time after B cell depleting therapy in patients in remission; T2). Cells were barcoded for patient and time, pooled, and stained with 38 antibodies conjugated to unique metal isotopes. Single-cell data acquired from time-of-flight mass cytometry (CyTOF) was clustered using Phenograph to identify cell clusters and how they evolved over time in each patient (see Methods). **(B)** Percentage of patients treated with prednisone and **(C)** average daily prednisone doses in relapsing and non-relapsing patients before anti-CD20 therapy (T0), in remission after immunosuppression withdrawal (T1), and at relapse (or at the same time point after anti-CD-20 therapy in non-relapsing patients; T2). **(D)** Percentage of patients treated with calcineurin inhibitors (CNI) at the same visits. n=30. *p < 0.05 *vs*. T0. ^#^p < 0.05 *vs*. relapsing at the same visit. Bar plots depict mean ± SEM.

Past analyses of B cell depleted patients relied on flow cytometry, which limited the scope of immune compartment analysis. We hypothesized that comprehensive and longitudinal peripheral immune cell analysis of reconstituted B and T cell subpopulations would confirm or identify new associations to predict/identify disease relapse post B cell depletion therapy. To address challenges of previous studies, we used time-of-flight mass cytometry (CyTOF) which utilizes heavy metal labeled antibodies, instead of fluorophores, to create immunophenotyping panels that are quantified by mass spectrometry. We analyzed and compared PBMC samples from steroid–dependent nephrotic syndrome (SDNS) patients who relapsed to those that did not after initially responding to B cell depletion (**Figure 1A**). CyTOF allows deconvolution analysis of over 40 distinct antibody markers to comprehensively characterize immune phenotypes. Using unbiased clustering, we assessed changes and tested for relationships with disease activities in (i) B cells subsets, ii) CD4^+^ and CD8^+^ T cell subsets, and iii) the major immune compartments.

## Methods

### Subjects and Sample Collection

We performed CyTOF analyses on frozen PBMCs serially collected from patients with SDNS maintained in remission with oral steroids and calcineurin inhibitors (CNI). SDNS was defined by two consecutive relapses during corticosteroid therapy tapering or within 14 days of steroid withdrawal that responded to the association of prednisone with cyclosporine or tacrolimus.

All the patients were in complete remission at enrollment and received a single dose of either ofatumumab (OFA, 1500 mg/1.73 m^2^) or rituximab (RTX, 375 mg/m^2^) infusion, as part of a randomized trial (NCT02394119 and Eudract.ema.europe.eu: 2015-000624-28) (19). After infusion, steroids were progressively tapered off until complete withdrawal, which happened within 3 weeks. At one week after complete steroid withdrawal, CNI were withdrawn within 3 weeks. For the present study, we included 17 patients who underwent a relapse of NS after full immunosuppression withdrawal and 13 controls who did not undergo relapse over the same follow-up period. PBMC were collected before OFA or RTX infusion (T0), during remission after immunosuppression withdrawal (T1), and at the time of relapse or at a similar time-point after OFA or RTX therapy in non-relapsing patients (T2). At T2, relapsing patients had already received steroid therapy to promote remission.

Complete remission was defined by urinary protein over creatinine ratio (uPCR) <200 mg/g (<20 mg/mmol) or 1+ protein on urine stick for 3 consecutive days (20). Partial remission was defined by proteinuria reduction of 50% or greater from the presenting value and absolute uPCR between 200 and 2000 mg/g (20-200 mg/mmol). NS relapse was defined as uPCR ≥2000 mg/g (≥200 mg/mmol) or ≥3+ protein on urine dipstick for 3 consecutive days.

The study was approved by the Institutional Review Board at the participating centers.

Study coordinator illustrated the project, delivered the information material and collected written informed consent from parents and child assent for treatment and collection of samples. Participants or their families could withdraw the consent at any time during the study.

### CyTOF Sample Preparation

To limit batch effect, we barcoded samples collected from the same subjects at 3 different times with anti-CD45 antibodies conjugated to unique metal isotopes before pooling the samples together. CyTOF sample preparation was conducted as previously reported by others (21). Antibodies were either purchased preconjugated from Fluidigm (formerly DVS Sciences, San Francisco, CA) or purchased purified and conjugated in-house using MaxPar X8 Polymer Kits (Fluidigm, San Francisco, CA) according to the manufacturer’s instructions. Ninety samples (30 subjects, 3 timepoints) were processed in 3 separate batches barcoded using 3 barcoding antibodies and pooled together. All PBMCs were stained with a panel of 38 antibodies (37 for clustering, 1 for viability) (**Supplementary Table 1**).

### CyTOF Data Acquisition

CyTOF data were acquired at Icahn School of Medicine at Mount Sinai as previously reported by others (21). Samples were acquired on a CyTOF2 (Fluidigm) equipped with a SuperSampler fluidics system (Victorian Airships) at a concentration of 1 million cells/ml in deionized water containing a 1/20 dilution of EQ 4 Element Beads (Fluidigm) and at an event rate of < 500 events/second. After acquisition, the data were normalized using bead-based normalization in the CyTOF software. Barcodes were demultiplexed using the Fluidigm debarcoding software. The data were gated to exclude residual normalization beads, debris, dead cells, and doublets, leaving live CD45^+^ events for subsequent clustering and high-dimensional analyses.

### CyTOF Data Analyses

We first clustered cells using the PhenoGraph algorithm (21) and we then curated the 20 metaclusters obtained. The frequencies for each common population were obtained by summation of the frequencies in each metacluster and subsequently debarcoded to obtain frequencies for each timepoint and patient. To minimize variability in measurement, our analysis strategy was structured as follows:

- Application of FlowSOM to computationally pooled samples: we pooled *in silico* all labeled cells from all timepoints together for each patient to analyze the major immune compartments (10,000 cells per timepoint for a total of 30,000 cells per patient), we applied FlowSOM, and then demultiplexed them. This allowed mapping of the same subsets in all the samples.
- Equal contribution of the samples: to avoid bias in FlowSOM for the subsets present in the samples with a greater number of CD4^+^ or CD8^+^ we maintained an equal contribution in the number of cells from every sample determined by the sample with the lowest number of CD4^+^ or CD8^+^ T cells.
- Reiteration: to increase power of the analyses for the cell subsets with low number of events, we reiterated the entirety of the sampling process and FlowSOM clustering up to 3 times to achieve robustness in the results.

Despite the level of stringency imposed by the repeated sampling and clustering process, we observed little dispersion in the results indicating that our findings were robust.

We did not observe significant differences in the average and SD of the signal for each marker nor in the frequencies of the major immune compartments across batches.

The number of CD45^+^ cells/mm^3^ was derived from measurements of the number of lymphocytes (T, B and NK cells) in each patient based on the frequencies of those major immune compartments.

### Cell Number Counts

Absolute cell number counts were obtained from total lymphocyte counts available from CBCs performed on these samples multiplied by the frequencies derived from the CyTOF analysis.

### Statistics

Statistical significance was determined by GraphPad Prism or R. Statistical tests used are reported in the figure legends. To establish all comparisons within relapsing and non-relapsing at different timepoints we performed a two-way ANOVA test, without assuming sphericity (Geisser-Green house correction) correcting for multiple comparisons by controlling the False Discovery Rate using the two-state step-up method of Benjamini and Yekutieli. Differences are considered significant at p ≤ 0.05.

## Results

### Baseline Patients’ Characteristics

We analyzed serial peripheral blood samples from 30 children with SDNS: baseline characteristics of included patients are shown in **Table 1**. At time of anti-CD20 therapy (before RTX or OFA; given at T0), all but one patient were receiving steroids and calcineurin inhibitors (**Figures 1B–D**). After receiving anti-CD20 therapy, patients underwent immunosuppressive withdrawal, and at T1 [after an overall median of 4.1 months (IQR 3.0 - 7.1); 3.0 (3.0-7.3) and 3.0 (3.0-7.0) months for relapsing and non-relapsing patients, respectively], all patients were still in complete remission with reduced or no immunosuppression (**Figures 1B–D**). During a median follow-up period of 7.2 months (IQR 6.0 - 11.8) (T2), NS relapse occurred in 17 patients [T2: 6.5 months (5.0-7.8)] who restarted steroids, while remission persisted in 13 [T2: 8.0 months (6.0-9.0)]. At the same visit (T2), non-relapsing patients were in remission with no immunosuppression (**Figures 1B–D**). There was no difference in relapse rates between RTX and OFA treated patients.

**Table 1:** Baseline characteristics of study participants.

	Total (n = 30)	Relapsing (n = 17)	Non-relapsing (n = 13)	P-value
***Demographics***				
*Age (yrs)*	11.6 ± 6.9	11.1 ± 6.7	12.3 ± 7.4	0.65
*Sex n (%)*				0.24
*Female*	6 (20)	2 (12)	4 (31)	
*Male*	24 (80)	15 (88)	9 (69)	
***Treatment***				0.29
*Rituximab n (%)*	15 (50)	10 (59)	5 (38)	
*Ofatumumab n (%)*	15 (50)	7 (41)	8 (62)	
***Prior rituximab use, n (%)***	12 (40)	6 (35)	6 (46)	0.36
***Number of prior relapses, n***	1.5 (1-10)	2 (1-10)	1 (1-6)	0.52
***Immunosuppressive drugs at enrollment (T0), n (%)***				
*Steroids*	30 (100)	17 (100)	13 (100)	1.0
*Tac*	16 (53)	9 (53)	7 (58)	0.96
*CsA*	13 (43)	8 (47)	5 (38)	0.63
***Labs***				
*Serum Creatinine (mg/dL)*	0.54 ± 0.24	0.53 ± 0.27	0.55 ± 0.20	0.85
*Serum Albumin (g/dL)*	3.73 ± 0.64	3.74 ± 0.55	3.71 ± 0.77	0.88
*Cholesterol (mg/dL)*	210 ± 61	192 ± 57	232 ± 61	0.09
*eGFR (ml/min)*	137 ± 38	142 ± 39	129 ± 37	0.35
*IgA (mg/dL)*	658 ± 231	638 ± 243	685 ± 221	0.59
*IgM (g/dL)*	166 ± 109	162 ± 127	171 ± 84	0.82
*IgG (mg/dL)*	144 ± 83	150 ± 99	134 ± 60	0.60
*Proteinuria (mg/24 h)*	99 ± 83	116 ± 104	78 ± 36	0.18

Data are mean ± SD number (%), or median (range). p-value compares the two treatment groups in a two-tailed t-test of heteroscedastic variance or chi squared. Tac, tacrolimus; CsA, cyclosporine.

### Changes in the Major Circulating Immune Compartments After B-Cell Depletion

**Figure 2:**
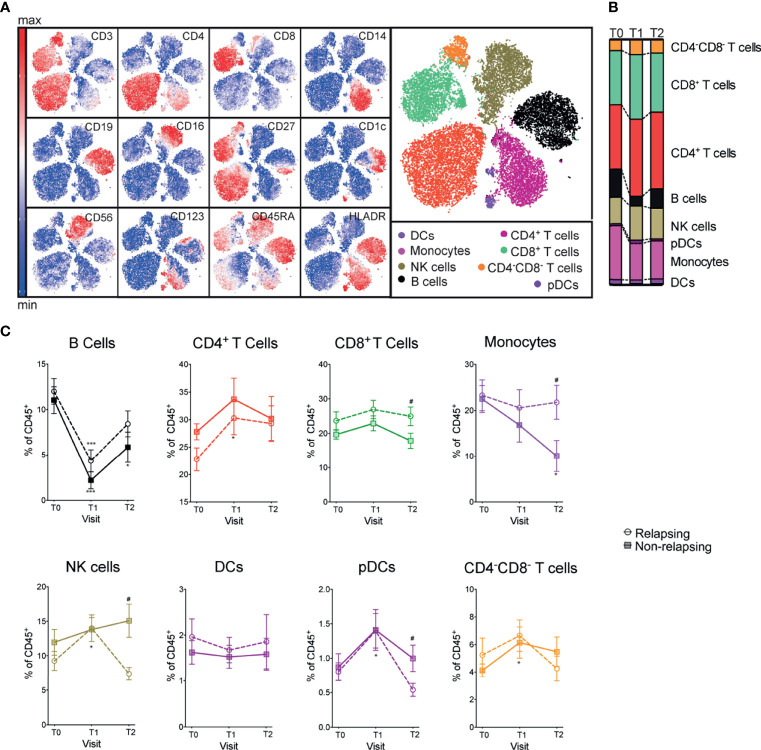
Frequencies of major immune compartments in INS patients receiving OFA or RTX therapy. **(A)** viSNE analysis of peripheral blood mononuclear cells (PBMC) from a representative patient including 3 time points (during immunosuppressive treatment, after immunosuppressive treatment withdrawal and at the time of relapse) colored by the relative expression of CyTOF markers to designate major immune clusters [populations defined in **(B)**]. **(C)** Percentages of major immune compartments once demultiplexed and based on the summation of Phenograph clusters before (T0) and serially after (T1 and T2) anti-CD20 therapy. Comparisons between relapsing and non-relapsing at the same timepoint ^#^p < 0.05. Comparisons between different timepoints *p < 0.05, ***p < 0.001. Two-way ANOVA corrected for multiple comparisons. Data points depict mean ± SEM.

We quantified and compared major immune compartment kinetics using unbiased clustering (Phenograph Clustering Algorithm (22), see Methods) with 12 markers and viSNE to visualize high-dimensional data in two dimensions while preserving single-cell resolution (23) (**Figure 2A**). B cell frequencies were significantly reduced after anti-CD20 treatment (OFA and RTX) and returned toward baseline values at T2 (**Figures 2B, C**). Frequencies of other major immune compartments, including macrophages, dendritic cells (DCs), plasmacytoid dendritic cells (pDCs), monocytes, NK cells, CD4^+^ T cells, CD8^+^ T cells and CD4^-^CD8^-^ T cells were not significantly affected by B cell depletion (visits T0 and T1; **Figures 2B, C**). At T2, relapsing patients had significantly decreased NK and pDCs and increased monocytes and CD8^+^ T cell frequencies compared to non-relapsing patients (**Figures 2B, C**).

### Proportion of Switched B Cell Subsets Is Higher in Relapsing Patients

**Figure 3:**
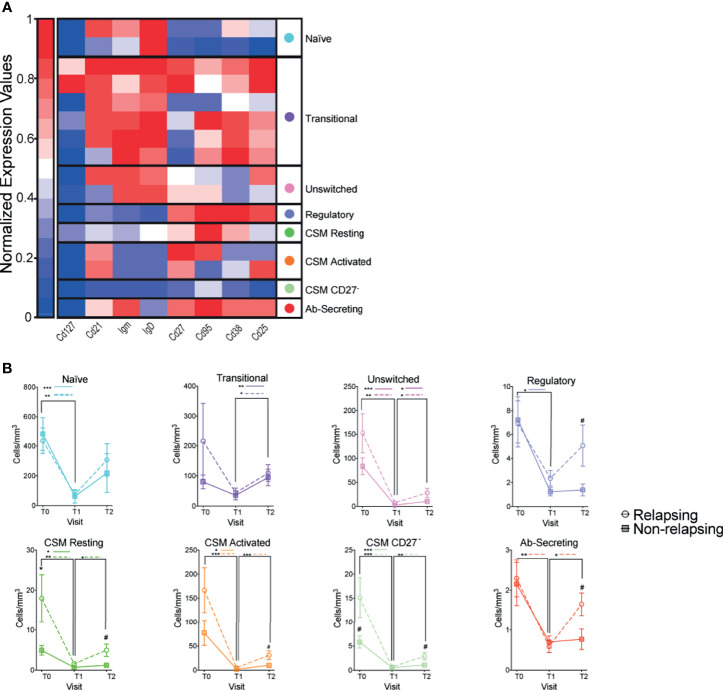
Changes in B cell subsets after anti-CD20 Ab therapy. **(A)** Heatmap of CD19^+^ immune cells colored and labeled by Phenograph cluster for all subjects and time points combined (see Methods). Rows represent different clusters identified and columns the relative expression of each marker in that particular cluster **(B)** Absolute numbers of CD19^+^ B cell subsets once demultiplexed and based on the summation of Phenograph clusters before (T0) and serially after (T1 and T2) anti-CD20 therapy. Comparisons between relapsing and non-relapsing at the same timepoint ^#^p < 0.05. Comparisons between different timepoints *p < 0.05, **p < 0.01, ***p < 0.001. Two-way ANOVA corrected for multiple comparisons. Data points depict mean ± SEM.

**Figure 4:**
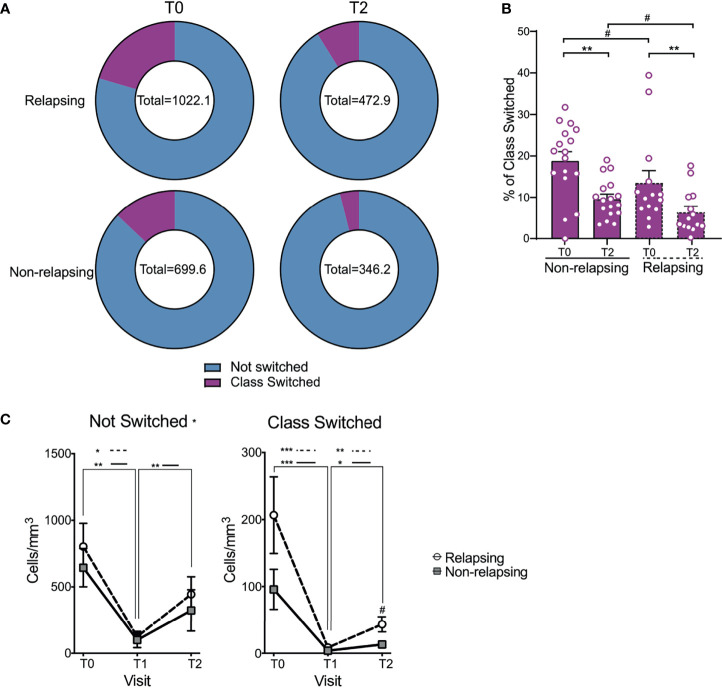
Changes in not switched and class switched B cells after anti-CD20 Ab therapy. **(A)** Doughnut pie graph of the number of class switched B cells (total = average number of class switched and not-switched cells per sample) before anti-CD20 treatment (T0) and at relapse (or at the same time point after anti-CD-20 therapy in non-relapsing patients) (T2). **(B)** Bar graph illustrating the percentage of class switched B cells. **(C)** Changes in absolute numbers of not switched and class switched B cells in relapse and non-relapsing patients at T0, T1 and T2. Comparisons between the relapsing and non-relapsing groups at the same timepoint ^#^p < 0.05. Comparisons between different timepoints *p < 0.05, **p < 0.01, ***p < 0.001. Two-way ANOVA corrected for multiple comparisons. Bar plots and data points depict mean ± SEM.

Magnitude of B cells (CD19^+^) decrease was similar between relapsing and non-relapsing patients (**Figure 2C**). Nadir B cell percentage in OFA-treated patients was significantly lower than in RXT-treated ones, but there was no significant difference between OFA and RTX in post-treatment B cell percentages or absolute numbers (**Supplementary Figures 1A, B**). We next compared kinetics and makeup of B cell subsets between relapsing and non-relapsing patients by performing a second level of unbiased clustering on B cells using 8 subset markers (CD127, CD21, IgM, IgD, CD27, CD95, CD38, CD25). B cells from all patients and all visits were pooled together, and clustered using B cell specific markers into subsets based on relative expression defining markers. Subsets were then demultiplexed to extract the subpopulation frequencies over time. We explored the different clusters using a heatmap displaying the relative expression of each marker per cluster and grouped them based on similarity (**Figure 3A**). Within the B-cell compartment we identified 10 distinct B-cell subpopulations, including B cells with a naïve (IgD^+^CD27^-^) (24), regulatory (CD25^hi^) (25, 26), antibody secreting (IgD^-^,CD27^+^,CD38^+^) (24), and memory phenotype (IgD^+^CD27^+^CD38^-^) (24). Frequencies of transitional B cells, B regulatory cells (B_REG_), and class-switched memory (CSM) resting B cells significantly increased after therapy (**Supplementary Figure 2**) despite global decreases in absolute numbers (**Figure 3B**). While we did not observe differences in single subset frequencies between the two groups at analyzed timepoints (**Supplementary Figure 2**), we found that frequencies of switched B cell subsets altogether (B_REG_, CSM resting, CSM activated, CSM CD27^-^, and Ab-secreting B cells) were significantly higher in relapsing patients than in non-relapsing individuals, both at T0 and T2 (**Figures 4A, B**, p<0.05). We also quantified composite absolute numbers of switched B cells and observed significantly higher numbers at T2 amongst relapsing patients (**Figure 4C**). At T1, there were no significant difference in switched B cells between individuals who fully withdrew immunosuppression and those who were on minimal residual immunosuppression (not shown).

### T Cell Subsets Are Not Different Between Relapsing and Non-Relapsing Patients

We next quantified and compared T cell subset frequencies over time between relapsing and non-relapsing patients. Several investigators have observed differences in T cell subpopulations after B cell depleting therapies that implicate a role in treatment effect (27, 28). We performed a second level of unbiased clustering within CD4^+^ and CD8^+^ T cell compartments using 18 (CD4^+^) and 17 (CD8^+^) subgroup markers, respectively (PD-1, TIGIT, ICOS, CD57, TIM3, Foxp3 only for CD4+, 2b4, CTLA4, OX40, CCR6, CXCR5, CD45RO, CD95, CD127, CCR7, CD25, CXCR3, CD45RA) quantifying subpopulation differences between study groups (**Supplementary Figure 3A** and **Supplementary Figure 4A**).

At T1, absolute numbers of CD4^+^ T cells, as well as central and effector memory subgroups, were lower in relapsing patients, but their frequencies were not different (**Supplementary Figures 3B, C**). Percentages of regulatory T cells (T_REG_) – a CD4^+^ T cell subset whose reduction others associated with relapse risk (29, 30) - did not significantly differ between groups. CD4^+^ T cells with a T-follicular helper-like (T_FH_) phenotype (see **Supplementary Figure 3** clusters for markers), a population of crucial importance in antibody production, were also similar between groups.

Unfractionated CD8^+^ T cell absolute numbers over time were similar between relapsing and non-relapsing patients (**Supplementary Figure 4C**). At T2, absolute numbers (but not percentages) of exhausted and effector memory (EM) CD8^+^ T cells were significantly higher in relapsing patients (**Supplementary Figures 4B, C**). At T1, there were no significant difference in CD4+ nor in CD8+ T cells between individuals who fully withdrew immunosuppression and those who were on minimal residual immunosuppression (not shown).

## Discussion

Our work is the first CyTOF-enabled immune compartment survey of INS patients receiving B cell depleting therapies. Standard flow cytometry is limited in the number of markers that can be probed in a single experiment due to autofluorescence and spectral spillover associated with fluorophores. CyTOF utilizes metal isotopes that possess unique mass spectrometry signatures enabling the analysis of over 40 cellular markers at the same time. Furthermore, CyTOF reduces experimental variability as metal isotopes can be used to tag samples with barcodes, allowing simultaneous sample analysis. Even with limited samples, CyTOF increases the likelihood that our results are reproducible, making them more clinically relevant.

A previous study in INS patients showed that accelerated reconstitution of CD19^+^CD27^+^IgM^-^IgD^-^ B cells (defined as switched-memory) identifies patients at higher risk of disease recurrence (31). Our data corroborates this specific finding in memory cells and extends such results using a more comprehensive characterization of B cell clusters. B cell depletion strategies are widely used in treating other autoimmune diseases. The present data in INS patients add to several published studies showing that recovery of class-switched memory B cells heralds relapses of other autoimmune diseases such as myasthenia gravis, neuromyelitis optica, and rheumatoid arthritis (32, 33). Therefore, monitoring relevant B cell subsets could guide timing of repeat treatment with depleting therapies or use of alternative immunosuppression regimens. This provides advantages over current clinical management that relies on markers of end organ damage – proteinuria and serum creatinine.

Intriguingly, switched B cells were significantly higher in relapsing patients despite the fact that T2 samples were taken after the initiation of steroid therapy, which has been shown to reduce this cell population (34, 35), a phenomenon that has been observed prior in INS (35) and other autoimmune conditions (36).

Five subsets of switched B cells recovered early in patients with disease relapse. In addition to class-switched memory cells, IgD^-^CD27^+^CD38^+^CD95^+^ Ab-secreting cells represent the subset most strongly associated with disease recurrence (37). This subpopulation lacks IgD expression and is CD27 positive, signifying mature B cells (38), and additionally express CD38. CD38 is a highly conserved type II glycoprotein that possesses pleiotropic effects on B-cell function and maturation (39–41). CD38 induces apoptosis in early B cells but promotes survival in germinal center B cells (42). Antibody-secreting cells, made up of plasma cells and immature plasmablasts (43), express high levels of CD38, whereas memory B cells, also IgD^-^ and CD27^+^ (44–46), lack CD38 expression (47, 48).

We also found that patients with disease relapse had a faster recovery of CD25^+^CD127^-^IgD^-^IgM^-^ B cells, a phenotype compatible with B_REG_. These cells inhibit T cells through the release of IL-10, IL-35, and transforming growth factor β (TGF-β) and their importance is known for several autoimmune conditions (49–51). Originally identified as a transitional B cell subset, it is now known that B_REG_ can acquire suppressive functions at different stages of development in response to environmental cues (52). Possibly, B_REG_ expansion in relapsing patients reflects a compensatory mechanism to active antibody production, similar to expansion of regulatory T cells (T_REG_) in kidney transplant recipients with acute rejection (53). However, more studies are needed to determine whether their presence is directly or indirectly related to INS pathogenesis. We analyzed T cell subpopulations because antibody mediated diseases (*e.g.* membranous nephropathy) depend on cognate B cell-T cell interactions. Previous studies showed that RTX affects homing of T_REG_ and T follicular helper cells (T_FH_) (54), possibly as a consequence of reduced interaction with B cells (55).

Most data on the efficacy of B cell depleting therapies in INS patients have been generated with rituximab, a chimeric anti-CD20 monoclonal antibody. Ofatumumab is a fully humanized anti-CD20 monoclonal antibody of last generation. OFA binds CD20 with more affinity, potentially leading to more efficient complement-dependent cytotoxicity (56, 57). In a small series, OFA-induced remission in children with SR-INS who did not respond to RTX (31). Our data confirm the higher peripheral B cell depleting efficacy of OFA compared to RTX. Despite this, kinetics of B cell reconstitution did not differ significantly between the two treatment arms suggesting that, at the doses used in our study, their effects on B cells in secondary lymphoid organs are similar. Because B cell reconstitution portended disease relapse, we hypothesized that differences in B cells subsets exist amongst patient groups.

B cell depleting agents have variable penetrance into secondary lymphoid structures. Relapse in patients with disproportionate persistence of antigen experienced B cells may represent preferential survival of autoreactive B cells. Consistent with this hypothesis, our data showed trends for increased frequency of Ab-secreting B cells that first occurred when B cells were depleted (T1, **Supplementary Figure 2**).

Our data provide basis to pursue critical questions regarding antigen specificity of surviving/reconstituting cell populations. The present findings are also consistent with murine data showing that podocyte targeting antibodies are sufficient to change the glomerular filtration barrier and are the “permeability factor” in some patients with INS due to FSGS (58). Clinically, our findings suggest that combined B cell therapies (*e.g.* RTX + proteasomal inhibitor) could provide enhanced treatment efficacy by targeting all class-switched and Ab-secreting cells.

B cells, especially the ones expressing activation marker CD25, can act as antigen presenting cells to activate T cells (59). However, in contrast to B cell subsets, our comprehensive immune phenotypic analysis identified few significant differences between relapsing and non-relapsing patients in CD4^+^ and CD8^+^ T cell clusters at T2. An exception to this were exhausted CD8^+^ T cells that were higher in relapsing patients. T cell exhaustion arises from prolonged antigen exposure (60) and is observed in patients with chronic autoimmune diseases (61) or renal transplant recipients (62). Higher frequencies of exhausted CD8^+^ T cells in relapsing patients at T2 raises the intriguing hypothesis that T cell exhaustion is a marker of disease chronicity/activity, indicative of increased B cell activation.

The main immune cell compartments were largely comparable between relapsing and non-relapsing patients with the exception of monocytes and NK T cells which underwent reciprocal changes. Changes we observed are likely related to resumption of high-dose corticosteroids in relapsing patients (63). Absence of differences in major immune compartments is an important negative finding with respect to B cell depleting therapies and emphasizes how CyTOF powered analysis provides uniquely in-depth and broad analysis.

### Limitations

Our study main limitation is the relatively limited sample size and the absence of prospective validation. Although the limitations associated with a small number of patients cannot be entirely overcome, CyTOF allowed comprehensive immune cell phenotyping with increased sensitivity for low-expressed markers. Therefore, we extracted a significant amount of information from a reduced number of samples. Additionally, we resampled and reanalyzed B-cell clusters 3 times to further ensure our findings were statistically robust and valid. Lastly, samples were run simultaneously, further decreasing variation due to technical issues.

Another intrinsic limitation of the study is the non-standardized time for blood sampling, as the time of relapses could not be planned in advance. However, the frequent sampling allowed us an excellent matching in the sample timing between relapsing and non-relapsing patients.

Previous studies have shown that RTX may affect not only B-cell composition, but also their function (64). Lack of functional studies on reconstituted switched B cells prevents any definitive conclusion on the effect of these cells in relapsing and non-relapsing patients. Future studies will be required to address this relevant issue as well as antigen specificity of reconstituted populations in relapsed patients. Our work provides the most solid basis, to date, for interrogating class-switched B cells in these patients.

## Conclusions

Overall, the present study demonstrates that 5 subsets of circulating switched B cells are preferentially increased in INS patients who relapsed after CD20-depleting therapies, suggesting a pathogenic link and potential for use as a biomarker. Our work provides focus and a strong rationale for future studies testing the hypothesis that monitoring the number and, potentially, function of switched B cells identifies patients at higher risk of relapse instead of using total B cells or clinical indicators, alone. Transcriptional and/or proteomic analyses of switched B cells in relapsing or non-relapsing patients may provide important mechanistic insights on INS pathogenesis.

## Data Availability Statement

The raw data supporting the conclusions of this article will be made available by the authors, without undue reservation.

## Author Contributions

PC, GG, MCo, and MF conceived and designed the study. MCi collected the samples. CC, JL, KB, and SB were involved in the preparation of samples for CyTOF analyses. MF performed all the statistical analyses. PC and MF critically interpreted the data and wrote the first draft of the manuscript together with LP. All authors contributed to the article and approved the submitted version.

## Conflict of Interest

The authors declare that the research was conducted in the absence of any commercial or financial relationships that could be construed as a potential conflict of interest.

## Publisher’s Note

All claims expressed in this article are solely those of the authors and do not necessarily represent those of their affiliated organizations, or those of the publisher, the editors and the reviewers. Any product that may be evaluated in this article, or claim that may be made by its manufacturer, is not guaranteed or endorsed by the publisher.
