# Supplementary material for: CyTOF-Enabled Analysis Identifies Class-Switched B Cells as the Main Lymphocyte Subset Associated With Disease Relapse in Children With Idiopathic Nephrotic Syndrome
Source: Front Immunol. 2021 Sep 21;12:726428. doi: 10.3389/fimmu.2021.726428 (PMC8490633; doi:10.3389/fimmu.2021.726428)
Supplement: Supplementary file 1 [file DataSheet_1.docx]

Supplementary Material

# Supplementary Tables

## Supplementary Table 1. CyTOF Markers.

| **Metal** | **Marker** | **Major Immune** | **B cells** | **T cells** |
| --- | --- | --- | --- | --- |
| In113Di | CD57 |  |  | X |
| Pr141Di | IgD |  | X |  |
| Nd142Di | CD19 | X |  |  |
| Nd143Di | CD45RA | X |  | X |
| Nd144Di | KLRG1 |  |  |  |
| Nd145Di | CD4 | X |  |  |
| Nd146Di | CD8 | X |  |  |
| Sm147Di | 2b4 |  |  | X |
| Nd148Di | CD16 | X |  |  |
| Sm149Di | CD127 |  | X |  |
| Nd150Di | CD1c | X |  |  |
| Eu151Di | CD123 | X |  |  |
| Eu153Di | PD-1 |  |  | X |
| Sm154Di | ICOS |  |  |  |
| Gd155Di | CD27 | X | X |  |
| Gd156Di | TIM3 |  |  | X |
| Gd158Di | CCR6 |  |  | X |
| Tb159Di | CTLA4 |  |  | X |
| Gd160Di | CD14 | X |  |  |
| Dy161Di | CD56 | X |  |  |
| Dy162Di | Foxp3 |  |  | X (only CD4+) |
| Dy163Di | CXCR5 |  |  | X |
| Dy164Di | CD45RO |  |  | X |
| Ho165Di | BAFFR |  |  |  |
| Er166Di | CD25 |  | X |  |
| Er167Di | CCR7 |  |  |  |
| Er168Di | CD3 | X |  |  |
| Tm169Di | OX40 |  |  | X |
| Er170Di | CD38 |  | X |  |
| Yb171Di | CD95 |  | X | X |
| Yb172Di | IgM |  | X |  |
| Yb173Di | CXCR3 |  |  | X |
| Yb174Di | HLADR | X |  |  |
| Lu175Di | TIGIT |  |  | X |
| Yb176Di | CD21 |  | X |  |
| Rh103Di | Viability |  |  |  |

# Supplementary Figures


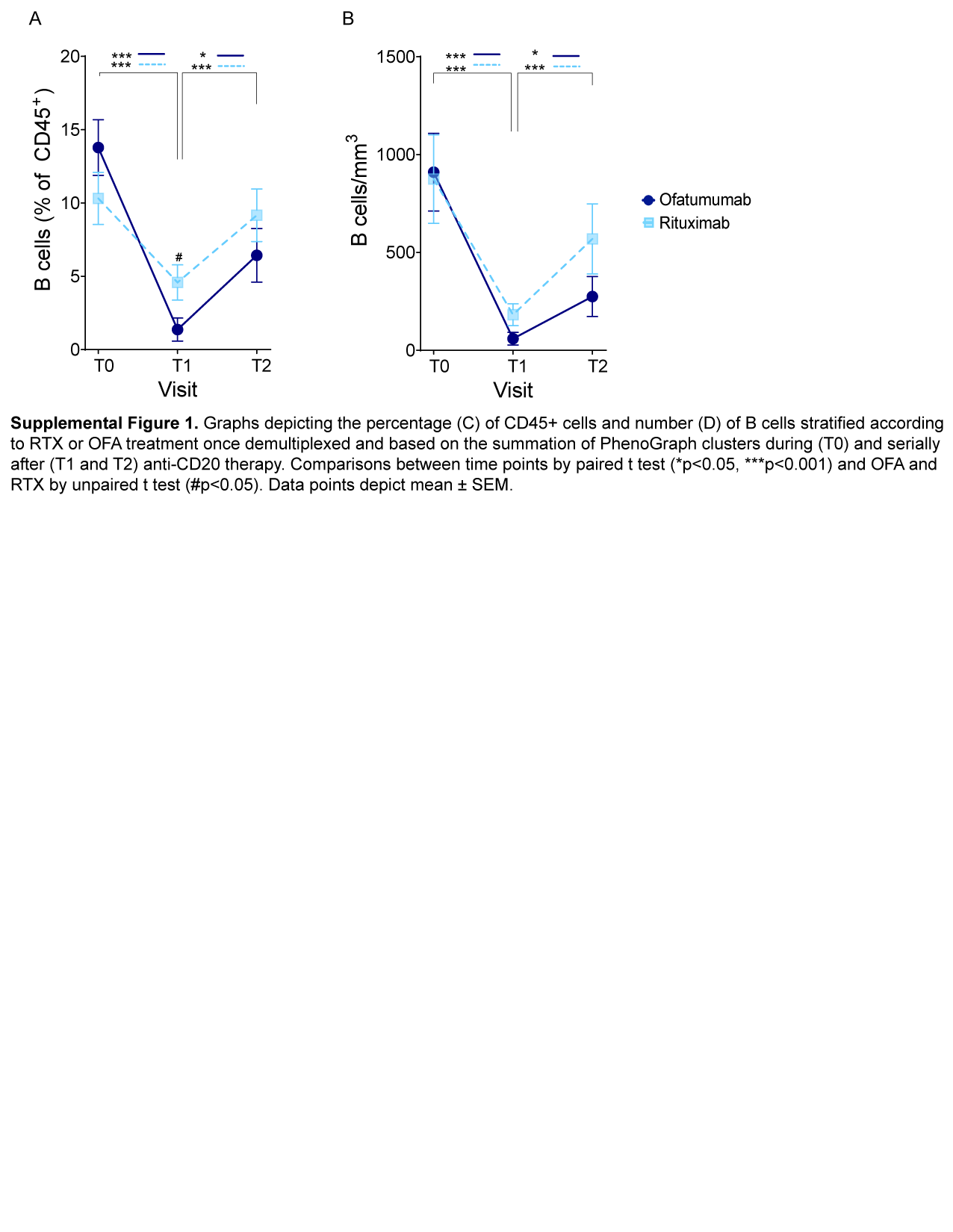


**Supplementary Figure 1. B cell depletion after anti-CD20 Ab therapy.** Graphs depicting the percentage of B cells (expressed as percentage of CD45^+^ cells) (**A**) and number of B cells (**B**) stratified according to RTX or OFA treatment once demultiplexed and based on the summation of PhenoGraph clusters before (T0) and serially after (T1 and T2) anti-CD20 therapy. Comparisons between RTX and OFA at the same timepoint #p<0.05. Comparisons between timepoints *p<0.05, **p<0.01, ***p<0.001. Two-way ANOVA corrected for multiple comparisons. Data points depict mean ± SEM.


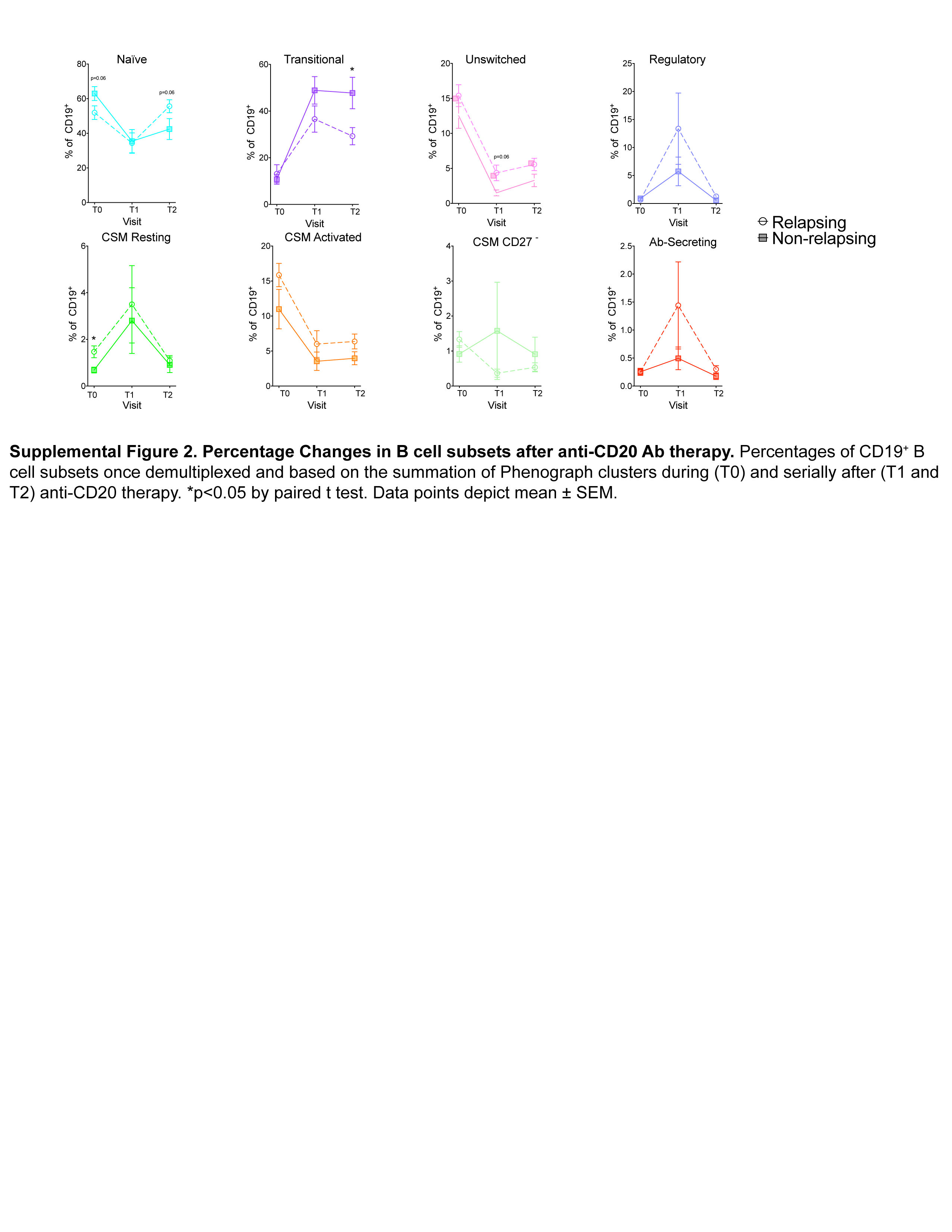


**Supplementary Figure 2. Percentage changes in B cell subsets after anti-CD20 Ab therapy.** Percentage of CD19^+^ B cell subsets once demultiplexed and based on the summation of PhenoGraph clusters before (T0) and serially after (T1 and T2) anti-CD20 therapy. Comparisons between relapsing and non-relapsing groups at the same timepoint were not significant. Comparisons between timepoints *p<0.05. Two-way ANOVA corrected for multiple comparisons. Data points depict mean ± SEM.

##
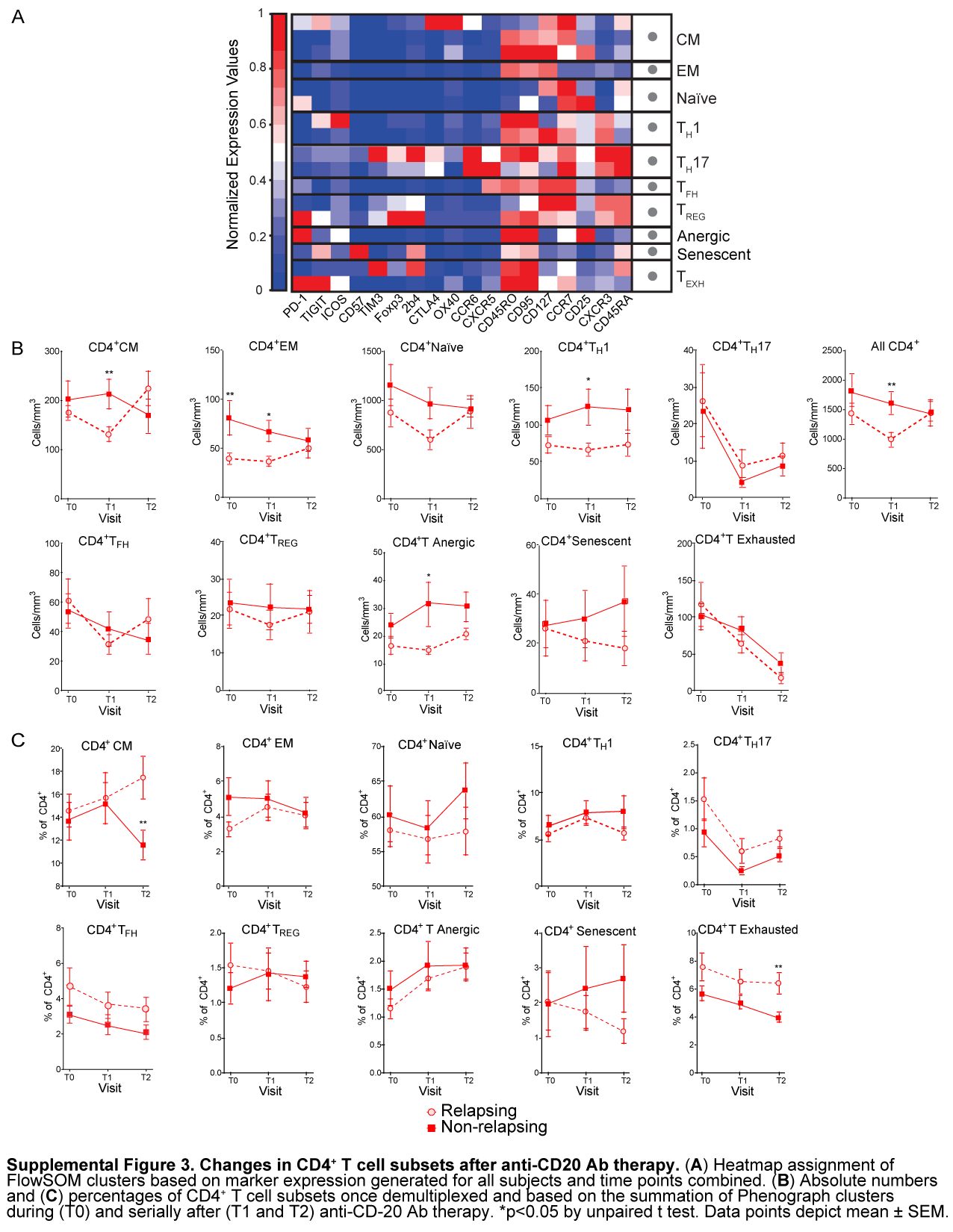


**Supplementary Figure 3. Changes in CD4^+^ T cell subsets after anti-CD20 Ab therapy.** Heatmap assignment of FlowSOM clusters based on marker expression generated for all subjects and time points combined (**A**). Absolute numbers (**B**) and percentages (**C**) of CD4^+^ T cell subsets once demultiplexed and based on the summation of PhenoGraph clusters before (T0) and serially after (T1 and T2) anti-CD20 therapy. *p<0.05 by paired t test. Data points depict mean ± SEM.


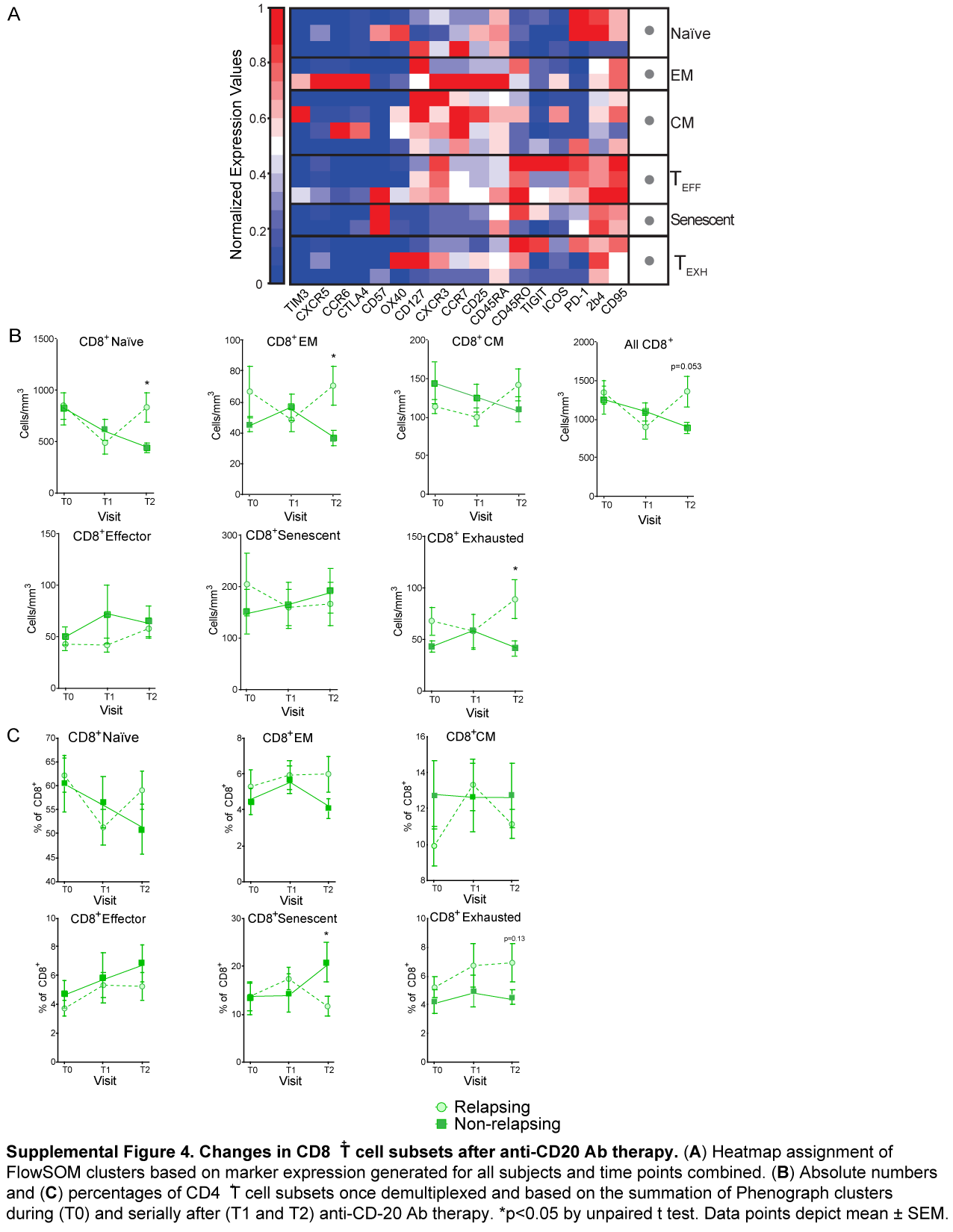


**Supplementary Figure 4. Changes in CD8^+^ T cell subset after anti-CD20 Ab therapy.** Heatmap assignment of FlowSOM clusters based on marker expression generated for all subjects and time points combined (**A**). Absolute numbers (**B**) and percentages (**C**) of CD4^+^ T cell subsets once demultiplexed and based on the summation of PhenoGraph clusters before (T0) and serially after (T1 and T2) anti-CD20 therapy. *p<0.05 by paired t test. Data points depict mean ± SEM.
